# Subclavian Artery Injury Following Central Venous Catheter Placement

**DOI:** 10.7759/cureus.14287

**Published:** 2021-04-04

**Authors:** Morris Sasson, Lisandro Montorfano, Stephen J Bordes, Mauricio Sarmiento Cobos, Mark Grove

**Affiliations:** 1 Vascular Surgery, Cleveland Clinic Florida, Weston, USA; 2 General Surgery, Cleveland Clinic Florida, Weston, USA; 3 Surgical Anatomy, Tulane University School of Medicine, New Orleans, USA

**Keywords:** catheter-related complications, jugular vein, subclavian artery, central venous catheter, vascular surgery

## Abstract

Mechanical complications following central venous catheterization are not uncommon. We discuss a case of iatrogenic intra-arterial central venous catheter placement requiring neck exploration in a 93-year-old woman. The catheter was inadvertently passed through the jugular vein and into the right subclavian artery by a junior surgical resident. Adequate technique and supervision, ultrasound guidance, and immediate diagnostic workup in the event of suspected arterial injury are factors necessary for physicians to minimize complications and provide safe medical treatment.

## Introduction

More than five million central venous catheterizations are performed in the United States each year of which more than 15 percent are expected to have complications [1-4]. There is significant literature assessing the safety and efficacy of central venous catheterization in general, but very few studies assess complications when the procedure is performed by a resident physician. As the number of residents continues to grow, these concerns become more evident. There are various types of complications. Infections and thrombosis are most prevalent and are closely followed by mechanical complications [5-7]. In this report, we describe a mechanical complication caused by a junior resident, which required neck exploration and removal of an intra-arterial catheter. The main goal of this case is to recognize areas in which training can improve in order to decrease complication rates. Training should always prioritize patient safety and excellent clinical outcomes.

## Case presentation

We describe the case of a 93-year-old woman who came to the ED with two days of syncopal episodes, altered mental status, and chest pain. The patient had a past medical history of hypertension, renal cell carcinoma requiring left nephrectomy, thyroid cancer requiring thyroidectomy, and subsequent hypothyroidism. The patient was evaluated by the Internal Medicine team and was found to be very weak and confused with a heart rate (beats per minute) ranging from the 120s to 130s. She also had episodes of sinus arrest and elevated troponins. She was admitted to the medical intensive care unit (ICU) with symptomatic sinus node dysfunction and tachycardia-bradycardia syndrome.

Upon admission, an ultrasound-guided right internal jugular catheter was placed without any reported complications or difficulties. Cardiology was consulted and the patient was started on an isoproterenol drip with plans for pacemaker placement. During the procedure, the central line was noted to be pulsatile and crossing the midline. Immediately after pacemaker placement, the Vascular Surgery team was consulted for evaluation and management of iatrogenic intra-arterial central catheter placement. 

**Figure 1:**
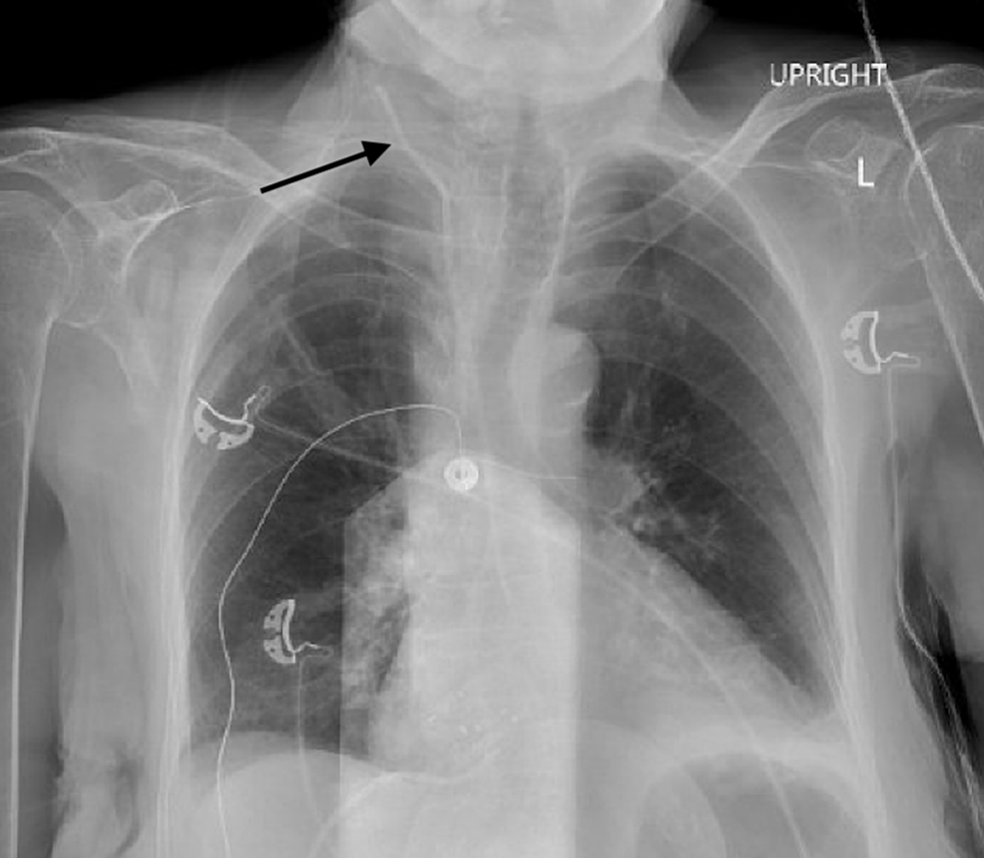
Upright chest X-ray showing the central venous catheter (arrow). L: left.

Upon vascular evaluation, a chest X-ray (CXR) was reviewed (Figure 1); and the central line was noted to have an unusual track with the tip projecting medially. To confirm the diagnosis of intra-arterial catheter placement, blood gases were obtained; and the line was evaluated for waveform and blood pressure. These studies showed clear arterial parameters confirming the presumed diagnosis. Ultrasound evaluation was inconclusive due to patient habitus. After discussing the risks and benefits of removing the line at the bedside versus in the operating room (OR), the patient was immediately taken to the OR.

**Figure 2:**
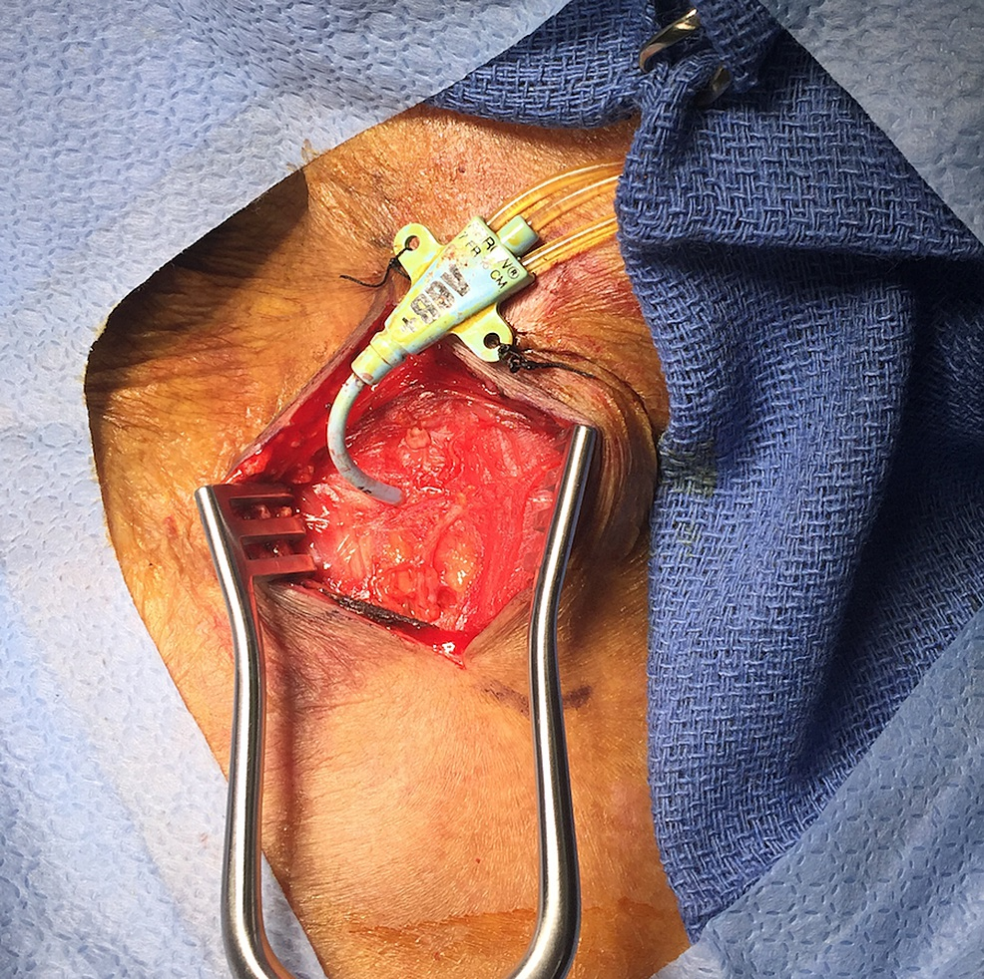
Right neck exploration showing the central venous catheter in place.

**Figure 3:**
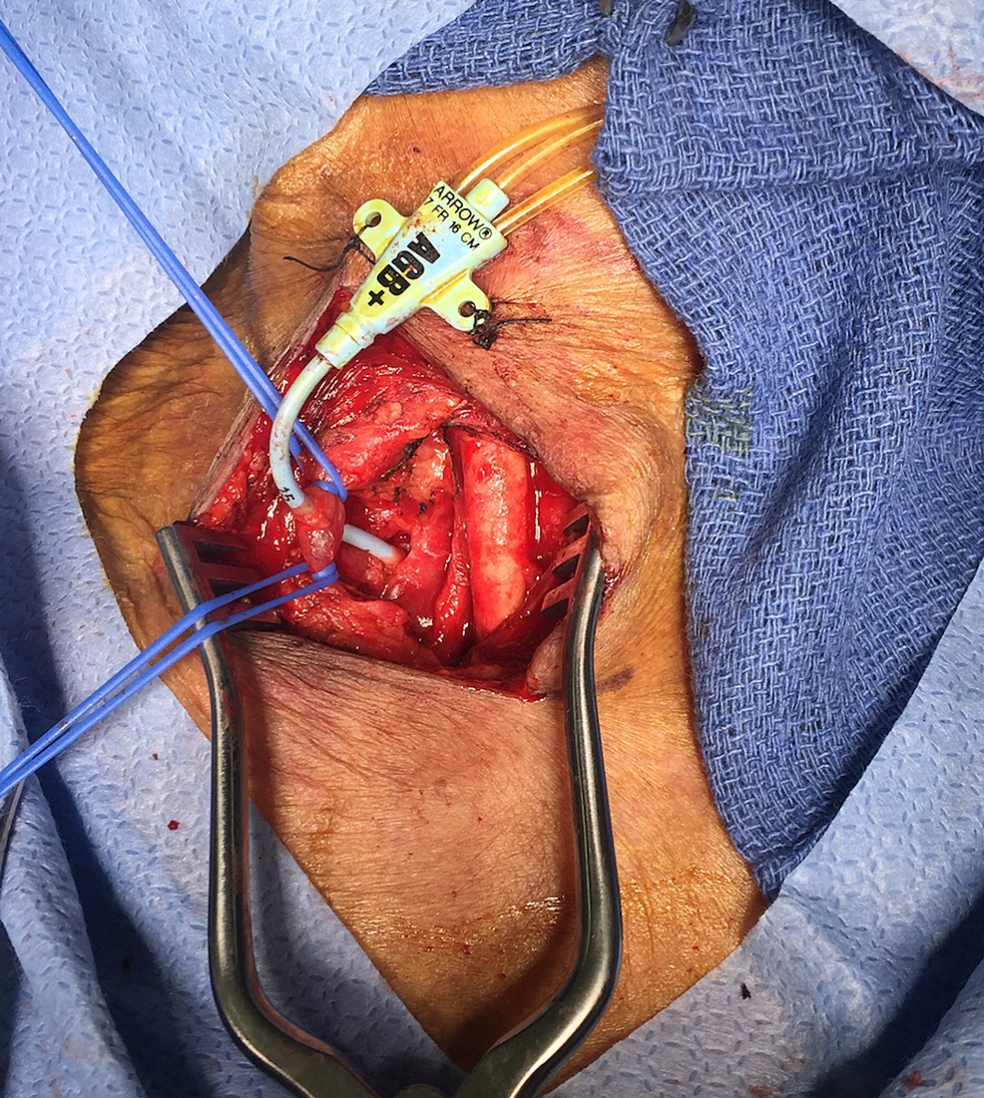
Catheter passing through the internal jugular vein (blue vessel loops) and entering the superior aspect of the right subclavian artery.

The patient was placed under general anesthesia and intubated. The patient's position was similar to that used for a carotid endarterectomy with the head slightly extended and turned towards the left. The catheter was left undisturbed and prepped in the operative field. An incision was made anterior to the sternocleidomastoid muscle (Figure 2). The right neck was explored. The jugular vein was encircled with vessel loops for proximal and distal control. The common carotid artery, which was felt to be the likely site of the arterial catheter, was in fact intact and free of disease. Unexpectedly, the catheter was noted to enter and exit the internal jugular vein. It did not injure the carotid artery but traveled deeper into the neck and chest, posterior to the right clavicle. The dissection was carried deeper and proximal to the clavicle. It was apparent that the catheter was entering the superior aspect of the right subclavian artery just lateral to the origin of the vertebral artery (Figure 3). The proximal and distal subclavian artery beneath the clavicle was mobilized sufficiently to encircle with a vessel loop. The vertebral and internal mammary arteries were identified and controlled with vessel loops. Under systemic anticoagulation, the subclavian artery was clamped proximally using a Martinez clamp; and the catheter was withdrawn from the puncture site. The entry site was closed using interrupted 5-0 polypropylene sutures. The jugular vein was repaired using interrupted 6-0 polypropylene sutures. A #10 Blake drain was placed, and the incision was closed in a standard fashion. Following surgery, the patient was transferred to the ICU, intubated.

On postoperative day (POD) 1, the patient was extubated. The neck incision was dry and without a hematoma. The drain had minimal output and was removed. On POD 2, the patient developed respiratory insufficiency requiring bilevel positive airway pressure (BiPAP). She had persistent altered mental status. The family requested Do Not Resuscitate (DNR)/Do Not Intubate (DNI) orders, but all other medical care was still provided. Neurology and Endocrinology teams were consulted, and the patient was diagnosed with metabolic encephalopathy as well as hypothyroidism with myxedema coma (thyroid-stimulating hormone [TSH]: 120 μU/mL). Treatment with intravenous levothyroxine was initiated. The Neurology team recommended further imaging evaluation with CT, MRI, and electroencephalogram (EEG); but the family declined further testing. On POD 5, the patient’s respiratory insufficiency and altered mental status worsened. The patient expired on POD 7.

## Discussion

Central line catheterization is a commonly performed procedure in the hospital setting; however, the level of experience of the physician performing the procedure is inversely proportional to the incidence of mechanical complications [7]. Insertion of 50 or more central lines has been shown to halve the incidence of complications [7]. It is important to emphasize that residents in training who place central venous catheters, mainly in the ICU, are in a learning phase. Direct supervision by a qualified physician who has met the aforementioned statistical criteria is a basic and fundamental measure to decrease the complication rates. Regular participation in surgical skills and trauma life support courses will further improve procedural skills. If a physician is unable to insert a central venous catheter after three attempts, a more experienced physician should take over as studies have shown that mechanical complications are six times more frequent after three or more attempts by the same provider [8]. Additional risk factors for complications include incorrect patient positioning, patients with prior surgery near the insertion site, deep vein thrombosis, high BMI, and coagulopathy, to name a few.

Mechanical complications associated with central catheter insertion can be limited with ultrasonographic guidance. Ultrasound allows physicians to identify the anatomy and measure the depth of the vein beneath the skin. This step helps to avoid injury to deeper structures. Under direct visualization, the needle is guided through the skin and introduced into the blood vessel. In facilities where ultrasound is accessible and physicians have the proper training, ultrasound guidance should be routinely used, especially for internal jugular venous catheterization. Ultrasound-guided subclavian venous catheterization remains controversial [9-14]. A CXR should be ordered in all patients following central line insertion to confirm the position of the catheter and rule out possible complications.

Injury to major arteries, such as the subclavian or carotid artery, can quickly become life-threatening, resulting in hemorrhage, ischemia, and stroke. Many of these vascular complications can be diagnosed at the bedside using point-of-care ultrasound [15]. If an arterial puncture occurs, pulsatile flow into the pressure transducer and bright red blood can be seen in most patients. However, in hypotensive or hypoxemic patients, these findings are not always evident. Blood gases can be drawn to confirm the diagnosis. In the case of a suspected or recognized arterial puncture, do not dilate the vessel. An experienced physician must be notified while removing the guidewire or needle and applying direct pressure to the artery. If the artery is dilated, immediate vascular surgery consultation is required for further management.

## Conclusions

This case details an iatrogenic subclavian artery catheterization as well as the steps taken to diagnose and treat the patient. Important points to consider in order to decrease complications of central venous catheter placement include adequate technique and supervision during the procedure, utilization of ultrasound, recognition of patient risk factors, and use of diagnostic methods to quickly identify suspected arterial injury. Physicians should recognize these issues to achieve the best possible patient outcomes and provide safe medical care.
